# Engagement Strategies to Improve Adherence and Retention in Web-Based Mindfulness Programs: Systematic Review

**DOI:** 10.2196/30026

**Published:** 2022-01-12

**Authors:** Natalie Winter, Lahiru Russell, Anna Ugalde, Victoria White, Patricia Livingston

**Affiliations:** 1 Centre for Quality and Patient Safety Research in the Institute for Health Transformation Deakin University Geelong Australia; 2 School of Nursing and Midwifery Deakin University Geelong Australia; 3 Centre for Quality and Patient Safety Research - Eastern Health Partnership Deakin University Melbourne Australia; 4 School of Psychology Faculty of Health Deakin University Geelong Australia; 5 Faculty of Health Deakin University Geelong Australia

**Keywords:** chronic disease, chronic illness, digital health, digital technology, internet mindfulness, mindfulness based stress reduction, patient dropouts, mobile phone

## Abstract

**Background:**

Web-based mindfulness programs may be beneficial in improving the well-being outcomes of those living with chronic illnesses. Adherence to programs is a key indicator in improving outcomes; however, with the digitization of programs, it is necessary to enhance engagement and encourage people to return to digital health platforms. More information is needed on how engagement strategies have been used in web-based mindfulness programs to encourage adherence.

**Objective:**

The aim of this study is to develop a list of engagement strategies for web-based mindfulness programs and evaluate the impact of engagement strategies on adherence.

**Methods:**

A narrative systematic review was conducted across the MEDLINE Complete, CINAHL Complete, APA PsycINFO, and Embase databases and followed the PRISMA (Preferred Reporting Items for Systematic Reviews and Meta-Analysis) guidelines. Articles were screened using the population, intervention, comparator, and outcome framework. Adults aged >18 years with chronic health conditions were included in the study. Mindfulness interventions, including those in combination with mindfulness-based cognitive therapy, delivered on the web through the internet or smartphone technology were included. Interventions lasted at least 2 weeks. Studies with a randomized controlled trial design or a pilot randomized controlled trial design were included. Engagement strategies, including web-based program features and facilitator-led strategies, adherence, and retention, were included.

**Results:**

A total of 1265 articles were screened, of which 19 were relevant and were included in the review. On average, 70.98% (2258/3181) of the study participants were women with a mean age of 46 (SD 13) years. Most commonly, mindfulness programs were delivered to people living with mental health conditions (8/19, 42%). Of the 19 studies, 8 (42%) used only program features to encourage adherence, 5 (26%) used facilitator-led strategies, and 6 (32%) used a combination of the two. Encouraging program adherence was the most common engagement strategy used, which was used in 77% (10/13) of the facilitator-led studies and 57% (8/14) of the program feature studies. Nearly two-thirds (63%) of the studies provided a definition of adherence, which varied between 50% and 100% completion across studies. The overall mean participant compliance to the mindfulness programs was 56% (SD 15%). Most studies (10/19, 53%) had a long-term follow-up, with the most common follow-up period being 12 weeks after intervention (3/10, 30%). After the intervention, the mean retention was 78% (SD 15%).

**Conclusions:**

Engagement strategies in web-based mindfulness programs comprise reminders to use the program. Other features may be suitable for encouraging adherence to interventions, and a facilitator-led component may result in higher retention. There is variance in the way adherence is measured, and intervention lengths and follow-up periods are inconsistent. More thorough reporting and a standardized framework for measuring adherence are needed to more accurately assess adherence and engagement strategies.

## Introduction

### Background

Mindfulness is the act of bringing awareness to the present moment in a nonjudgmental and accepting way [1]. Mindfulness programs are increasing in popularity as nonpharmacological alternatives to manage both physiological and psychological outcomes related to health conditions [2]. Psychological benefits are evident in individuals across a variety of conditions, including cancer [3] and mental illness [4], and physical health outcomes have been observed through improved blood pressure control [5] and improved glycemic control in people living with diabetes [6].

Evidence shows that mindfulness skills can be improved through greater engagement with meditation, home practice, face-to-face contact with a facilitator, and a higher number of sessions per week [7]. High adherence to both face-to-face and web-based mindfulness programs results in significant improvements in well-being outcome measures [8,9].

Mindfulness programs are increasingly being adapted to web-based platforms, providing opportunities for more people to participate compared with conventional face-to-face sessions [10]. Typically, adherence to web-based interventions is low, both with program adherence and study attrition [11]. Program adherence is poorly defined but needs to be standardized across studies; however, it is commonly conceptualized by the number of log-ins or number of sessions or modules completed in a program [12].

Adherence to web-based programs in previous reports has varied between 39.5% and 92% [9] compared with adherence to face-to-face settings, where the rates ranged between 26% and 100% [13] (based on definitions of 100% program completion). Mindfulness programs are often 8 weeks long in duration [9], with higher adherence having an impact on improved participant outcomes [14]. There is a need to explore whether engagement strategies can improve adherence to unmoderated web-based interventions. High attrition in telehealth interventions is common and can undermine the potential impact of programs [15]. Adherence to mindfulness-based interventions is often poorly defined and inconsistent across studies [16]. Promoting long-term adherence and engagement with web-based interventions may maximize the potential outcomes [17].

Engagement refers to the frequency and duration of use of the interventions, such as logging in and out of programs [18]. Strategies to support engagement are used to encourage and draw people back to the interventions [18]. Engagement can be enhanced by the design and features of web-based interventions, including the use of gamification, breaking content into manageable blocks, and using a variety of formats to deliver content such as video and visuals [19]. Other considerations for improving engagement include guided interaction from trained personnel [18], asynchronous emails [20], or web-based features such as reminders [18]. Behavior change techniques are engagement strategies incorporated into interventions to promote sustainable changes in behavior [21]. Behavior change techniques, such as notifications and semiautomated tracking, have previously been adopted in app-based interventions and have shown a positive impact on improving engagement [22]. In mindfulness programs, engagement involves regular meditation and daily awareness exercises coupled with intention motivation and commitment to practice [23]. Techniques such as self-reflection are incorporated into mindfulness programs and have been shown to positively impact symptoms in people with anxiety and stress [24]. More recent techniques such as machine learning [25] may also be used to tailor interventions to user-specific needs, thereby maximizing the clinical outcomes of users.

The influence of engagement strategies on program adherence has not been compared across studies; however, it is an important consideration when designing and implementing web-based interventions. In this review, we explored the engagement strategies applied in web-based mindfulness programs and evaluated whether these strategies had an impact on program adherence and retention.

### Research Question

The following research question was used in the study: how can engagement strategies be incorporated into web-based mindfulness programs to improve adherence and retention?

### Objectives

The objectives of this study are (1) to develop a list of engagement strategies for web-based mindfulness programs and (2) to evaluate the impact of engagement strategies on adherence.

## Methods

### Search Process

This systematic review was guided by the PRISMA (Preferred Reporting Items for Systematic Reviews and Meta-Analysis) framework [26]. The following databases were searched for terms related to mindfulness, web-based programs, and engagement strategies: MEDLINE Complete, CINAHL Complete, APA PsycINFO, and Embase. The literature search focused on identifying papers published between January 2015 and March 2020. A 5-year period was chosen to capture the most recent web-based interventions. See Table S1 in Multimedia Appendix 1 for an example of the search strategy applied to the MEDLINE database. The reference lists of relevant articles and systematic reviews were searched for additional articles.

### Eligibility Criteria

To guide the eligibility and screening process, the PICO (population, intervention, comparator, and outcome) framework [27] was used:

#### Population

Adults aged ≥18 years with a diagnosed chronic health condition or self-reported anxiety or depression were included in the study.

#### Intervention

Mindfulness interventions delivered on the web through the internet or smartphone technology were included. Mindfulness programs were defined as those focusing specifically on mindfulness-based practice, including programs using a combination of mindfulness and cognitive behavioral therapy (mindfulness-based cognitive therapy).

To allow for engagement strategies and adherence to be analyzed, the interventions had to be at least 2 weeks in duration. There is limited research to describe how long interventions should be to warrant the inclusion of engagement strategies. Previously, engagement was measured by reflecting on the previous 2 weeks [23]. Therefore, we determined that interventions had to be at least two weeks in duration to be included in the review.

#### Comparator and Context

Studies were required to have a comparison group with a randomized controlled trial (RCT) or a pilot RCT design.

Mindfulness programs developed by research groups for specific populations or commercially available mindfulness programs were tested in controlled trial settings.

#### Outcomes

Program adherence, study retention rate (%), and strategies such as web-based program features and facilitator-led features were included.

### Screening

Retrieved articles were uploaded and managed by Endnote X9 (Clarivate Analytics). Duplicates were removed, and titles and abstracts were screened by 1 author (NH). Full-text articles were uploaded to *Covidence* to allow cross-checking between authors [28]. Full texts were reviewed independently by 2 authors, and any disagreements were resolved through discussion.

### Data Extraction

A data extraction tool was developed in Microsoft Excel to standardize the extraction. Data were extracted by 1 author (NH), and 10% were cross-checked by the second author (PL).

#### Study Characteristics

Study data including author, year of publication, country, design, number of participants, intervention type, intervention duration, follow-up measurements, prior mindfulness experience, recruitment method, financial compensation, commercial app name, primary outcome, and primary findings were extracted.

#### Participant Characteristics

Gender, age, race, ethnicity, type of chronic illness or condition, and patient and caregiver status were extracted.

#### Adherence

Studies were included in the review when they reported per-protocol and intention-to-treat analyses. Because of variance in reporting the intervention, adherence was assessed in 3 different ways depending on the data available:

1. As a percentage of compliance with the intervention protocol. For example, some authors defined adherence as 80% program completion, and in this review, we recorded the percentage of the sample that was adherent with 80% program completion.
2. In groups defined by the study authors. For example, in an 8-week program, some authors reported the percentage of people who were adherent with 0- 3 sessions, 4- 6 sessions, and 7-8 sessions. In this review, we recorded the percentage of the sample that was adherent with the highest group of completion, for example, 7-8 sessions.
3. Summarized findings of the frequency and duration of use.

#### Retention

Retention rates were reported for the intervention group at postintervention measurements and subsequent follow-up points.

#### Engagement Strategies

Engagement strategies were categorized into following 3 groups:

1. Program features, including chat rooms, discussion boards, diaries and reflective processes, automated reminders, social support, goal setting, mood tracking, customization of content, demonstrations of meditation practice, and immediate feedback on meditation practice;
2. Facilitator-led strategies, including reminders from the research team to continue practice, contact with the research team to discuss practice or monitoring, and response to well-being scores throughout the intervention; and
3. A combination of program features and facilitator-led strategies.

### Data Analysis

Study characteristics, participant characteristics, adherence, and retention rates were analyzed using descriptive statistics.

Data analysis consists of the following:

1. Exploring adherence: how adherence was defined, the impact of adherence on outcomes, impact of financial compensation on adherence, and impact of intervention length on adherence.
2. Describing retention at postintervention measurements and the last data collection point.
3. Describing engagement strategies (program features, facilitator-led strategies, or a combination): engagement strategies were categorized and summarized using frequency statistics.
4. Assessing the impact of engagement strategies on adherence: the relationship between engagement strategies and adherence was analyzed by comparing the type of engagement strategy (program features, facilitator-led strategies, or a combination) with the percentage of people who reached program adherence or the percentage of people who adhered with the highest group of sessions (eg, those who completed 7-8 sessions in an 8-week program, as defined by the study authors).

- Assessing the impact of engagement strategies on retention: the relationship between engagement strategies and retention was measured by comparing the type of engagement strategy with the intervention length, retention at the postintervention measurement and retention at the last follow-up points.

## Results

### Study Characteristics

**Figure 1:**
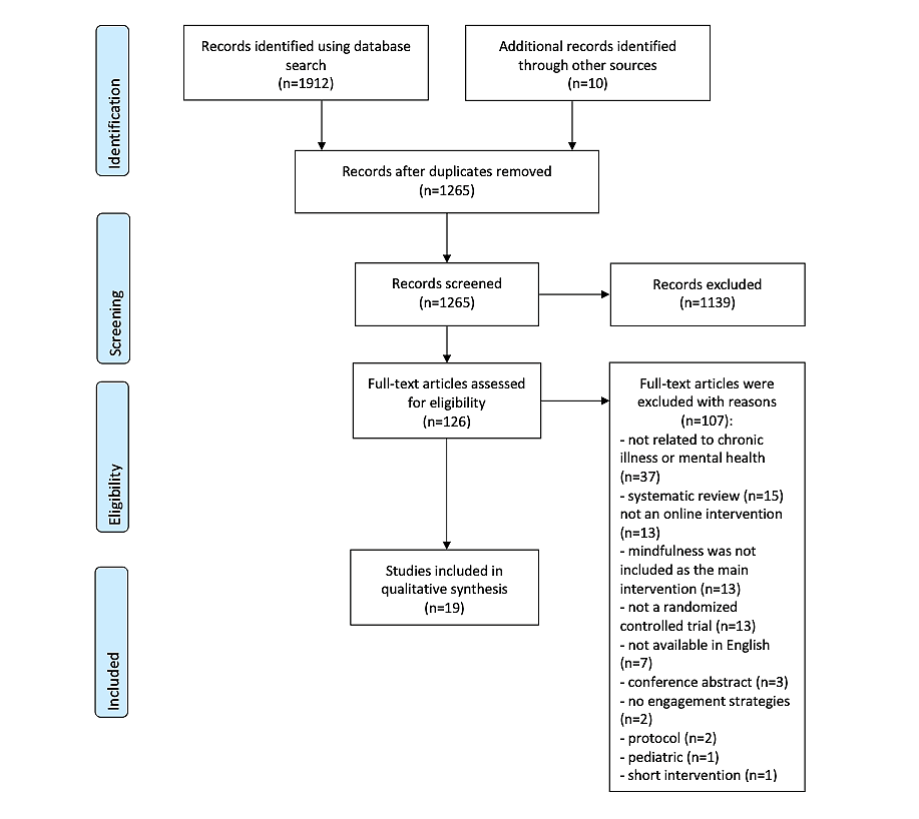
PRISMA (Preferred Reporting Items for Systematic Reviews and Meta-Analysis) diagram of the search process.

A total of 1922 articles were retrieved from the databases and reference lists. After removing duplicates, a total of 1265 articles were screened by title and abstract. Full texts were retrieved for 126 articles, of which 19 were included in the review (Figure 1). Most studies were conducted in the United States (9/19, 47%) [5,24,29-35], were RCTs (16/19, 84%) [5,14,24,29,31-33,35-43], web-based (11/19, 58%) [3,14,33-35,37-43], and focused specifically on mindfulness or meditation (15/19, 79%) [3,5,14,29-32,34-36,39-43] (Table S2 in Multimedia Appendix 1) [3,5,14,24,27,29-41,43]. More studies (10/19, 52%) excluded people with previous or recent mindfulness experiences [24,29-31,37,39,42,43] than those who allowed participants with prior mindfulness experience (6/19, 31%) [3,14,32,34,35,38,40,41]. Over half of the studies used a combination of web-based and face-to-face recruitment strategies (10/19, 52%) [5,29,31,32,34,35,37,39,40,42]. Commercially available mindfulness apps, including Headspace (n=3) [30,32,36], Calm (n=1) [29], and Pacifica (n=1), were used by 5 (26%) studies [24]. A total of 3 (16%) studies provided monetary compensation for participation [29,30,33], and 3 (16%) provided access to paid mindfulness apps [30,32,36]. Intervention duration ranged from 2 weeks to 12 months, with over half (10/19, 53%) of the studies having an intervention duration of 8 weeks [29,30,32,33,36,37,39,40,42,43].

A total of 8 (47%) studies focused on psychological measures as their primary outcome [29,31-34,37-39,42], and 3 (16%) used a physiological measure [5,14,41]. A total of 7 (37%) studies did not report the primary outcome [3,24,30,35,36,40,43]. Secondary outcomes were predominately psychological measures and program evaluations (8/19, 42%) [16,24,30,32,34,38,39,42]. Most (17/19, 90%) studies showed that mindfulness resulted in a significant improvement in outcomes either psychological or physical [3,5,14,24,29-34,36,37,39-43] (Table S3 in Multimedia Appendix 1) [3,5,14,27,29-41,43].

### Participant Characteristics

A total of 34,601 participants were included in the trials. The mean sample size was 165 (SD 134; range 21-500). On average, 71% of the participants were women (SD 18; range 46-100) and 46 years old (SD 13; range 21-76). A total of 8 (42%) studies reported the ethnicity of the participants [24,29-35,39], and 4 (26%) reported race [29,31-33]. On average, White people comprised 74% (SD 14%) of the sample and 90% (SD 10%) were non-Hispanic. Mindfulness programs were delivered to people with a variety of chronic illnesses, with the most common conditions related to mental health (8/19, 42%) [24,29,31,33-36,38] and cancer (4/19, 21%) [3,30,32,37]. Most (17/21, 81%) studies were delivered to people living with the illness [3,5,14,24,29,31-41,43].

### Engagement Strategies

A total of 8 (42%) studies used only program features to encourage adherence, 5 (26%) used only facilitator-led strategies, and 6 (32%) used a combination of the two (Table 1).

Within the facilitator-led strategies (n=13) [5,30,31,33-38,42,43], encouraging adherence was most commonly done using contact and reminders from facilitators to use the program (10/13, 77%) [30,31,33-36,38,42]. Contact with a facilitator for discussion of content or well-being scores was used to a lesser extent (4/13, 31%) [5,37,38,43]. In 7 (37%) studies, engagement with facilitators occurred weekly [34-37,42,43].

**Table 1:** Types of engagement strategies used across studies and their adherence rates.

Study	Program engagement	Facilitator engagement	Adherence with study protocol (%)
Chandler et al [5]	✓	✓	39
Compen et al [37]	✓	✓	79
Kladnitski et al [38]	✓	✓	66
Kubo et al [30]	✓	✓	56
Stjernsward and Hansson [27]	✓	✓	57
Thompson et al [33]	✓	✓	NR^a^
Gotink et al [14]	✓	N/A^b^	50
Hearn and Finlay [39]	✓	N/A	72
Henriksson et al [40]	✓	N/A	58
Huberty et al [29]	✓	N/A	NR
Moberg et al [24]	✓	N/A	NR
Rosen et al [32]	✓	N/A	NR
Russell et al [3]	✓	N/A	NR
Younge et al [41]	✓	N/A	53
Bostock et al [36]	N/A	✓	27
Lindsay et al [31]	N/A	✓	NR
Tavallaei et al [43]	N/A	✓	NR
Wahbeh et al [35]	N/A	✓	NR
Wahbeh [34]	N/A	✓	NR

^a^NR: not recorded.

^b^N/A: not applicable.

Within program feature strategies (n=14) [3,5,14,24,29,30,32,33,37-42], participants in 57% (8/14) studies received automated reminders [3,5,14,29,30,32,40,41]. Half of the program reminders were received at least once a week [3,14,32,41], and the remaining were sent on an ad hoc basis [29,30] or participants were able to personalize whether they received notifications or not [5,30]. Other program features used to encourage adherence included the ability to personalize mindfulness course content (4/14, 29%) [5,25,26,28], homework activities (3/14, 21%) [33,37,38], self-reflections (2/14, 14%) [37,42], social contact (3/14, 21%) [5,24,33], personalization of app appearance (2/14, 14%) [5,24], lesson summaries (1/14, 7%) [38], progress tracking of mindfulness practice (1/14, 7%)[24], immediate feedback on practice (1/14, 7%) [5], demonstration videos (1/14, 7%) [39], goal setting (1/14, 7%) [24], tracking psychological outcomes (1/14, 7%) [24], and tracking physical health (1/14, 7%) [24] (Table S4 in Multimedia Appendix 1) [3,5,14,24,29-35,37-41,43].

Contact initiated by facilitators or program reminders was most commonly delivered by email (9/14, 64%) [3,14,33,36-38,40-42] or telephone (7/14, 50%) [30,31,33-35,38,43].

### Adherence

Nearly two-thirds (12/19, 63%) of the studies provided a definition of program adherence [3,5,14,30,33,36-42]. When defined as the percentage of program completion, the definitions of adherence varied between 50% and 100% program completion across studies. When adherence was grouped, the highest group of completion varied from 50% to 100% among the studies. A total of 6 (32%) studies did not provide a measurement for adherence and analyzed program use descriptively [24,29,31,32,34,35]. Moreover, 1 (5%) study did not report adherence or program use [43]. The percentage of people who complied with the authors’ definitions of adherence ranged from 27% to 79%, with a mean compliance of 56% (SD 15%).

### The Impact of Engagement Strategies on Adherence

Among studies that used only program features (n=8) [3,14,24,29,32,39-41], 4 recorded adherence between 50% and 72% (mean 58%, SD 8%) [14,39-41] (Table S3 in Multimedia Appendix 1). Among studies that used only facilitator-led strategies (n=5) [31,34-36,43], only 1 reported adherence of 27% [36]. Among studies that used a combination of program features and facilitator-led strategies (n=6) [5,30,33,37,38,42], 7 recorded adherence between 39% and 79% (mean 59%, SD 13) [5,30,37,38,42].

When examining studies that used program features, of the studies that used 1 strategy (n=6) [3,14,39-42], 5 measured adherence rates between 50% and 72% (mean 58%, SD 8%). Of the studies that used 2 strategies (n=8) [29,30,32,33,37,38], 3 measured adherence between 56% and 79% (mean 67%, SD 9%). A total of 5 studies did not include any engagement strategies within their program [15,34-36,43], and 2 [5,24] used ≥5 strategies; adherence was only recorded in 2 of these studies, and they were below 40%. Studies that involved only program reminders as engagement strategies (n=4, 3 studies recorded adherence) [3,14,40,41] had an average adherence rate of 54% (SD 3%) compared with the average adherence rate of 48% (SD 8%) of those studies that used reminders and other strategies (n=4, only 2 recorded adherence) [5,29,30,32], and the average adherence rate of 69% of those studies that did not use program reminders but only used other strategies (n=6, 4 recorded adherence) [24,33,37-39,42].

### How Adherence Affected Outcomes

A total of 10 (53%) studies analyzed the relationship between outcome variables and adherence [14,24,30,32,34,36,38-40,42]. Of them, 4 studies found that people who had higher adherence to mindfulness programs had a significantly higher improvement in outcomes [30,36,40,42]; 1 study found that people with higher scores for depression at baseline were less likely to be adherent or complete mindfulness programs [39]; 1 found that people with higher blood pressure readings were more likely to be compliant [14]; and 1 showed that higher quality of life scores at baseline were significantly associated with improved adherence [32]. A total of 3 studies found no relationship between baseline scores and adherence or adherence and outcome variables [24,34,38].

### Financial Compensation and Program Adherence

Of the 6 studies that provided any type of compensation, 2 measured adherence with a mean of 42% (SD 15%; range 27-56) [30,36]. Among the studies that did not offer financial compensation, the majority (8/13, 62%) measured adherence with a mean of 60% (SD 11%; range 39-79) [5,14,37-42].

### Intervention Length and Program Adherence

The impact of the intervention length on adherence was analyzed. Of the 5 studies with an intervention <8 weeks, none recorded adherence. Those with an 8-week intervention recorded an average of 58% (SD 16%) adherence (6/10, 60% of the studies measured adherence) [30,36,37,39,40,42]; those with interventions >8 weeks recorded an average of 52% (SD 9%) adherence (4/4, 100% of the studies measured adherence) [5,14,38,41].

### Retention

Most (10/19, 53%) studies conducted pre-post analysis with additional follow-up points [14,24,29,32-34,36,38,39,42]. Follow-up periods ranged from 4 to 36 weeks after intervention, and the most frequent follow-up time was 12 weeks after the intervention (3/10, 30%) [38,39,42]. After intervention, most (14/19, 74%) studies had over 70% retention (mean 78%, SD 15%; range 35%-100%) [3,5,29-31,33-39,41,43]. At the last follow-up point, 4 studies had retention above 70% [14,33,36,38].

### The Impact of Engagement Strategies on Retention

Studies that applied only facilitator-led strategies, on average, were 6 weeks in duration (SD 2; range 2-8) and had a retention rate of 93% (SD 10; range 73-100) compared with studies with a combination of program features and facilitator-led strategies with a mean duration of 16 weeks (SD 10; range 8-52) and a retention rate of 75% (SD 5%; range 69-84) and those with only program features with a mean duration of 8 weeks (SD 2; range 4-12) and retention rate of 67% (SD 15%; range 30-79).

Of the studies that used facilitator-led strategies only, 40% (2/5) had follow-up periods after postintervention follow-up [34,36]. On average, follow-up was 7 (range 6-8) weeks and retention was 76% (SD 15%; range 69-82). Of the 6, 5 (50%) studies using a combination of program features and facilitator-led strategies had long-term follow-up, which, on average, was 11 weeks (SD 0.9; range 10-12), with a retention rate of 71% (SD 15; range 49-83) [33,38,42]. Of the 8, 5 (63%) studies using program features only also had a long-term follow-up period of, on average, 13 (range 4-36) weeks, with retention rates of 53% (SD 18; range 20-74) [14,24,29,32,39].

Studies that used only program reminders as engagement strategies (n=4) [3,14,40,41] had mean retention rates of 71% after intervention (n=3) [3,40,41] and retention of 74% at the last follow-up point (n=1) [14]. Studies that used reminders and other strategies (n=4) [5,29,30,32] had a mean retention of 78% after intervention (n=3) [5,29,30] and 57% at the last follow-up point (n=2) [29,32]. Studies that did not use program reminders but only used other strategies (n=8) [24,33,37-39,42] had a mean retention of 67% after intervention (n=6) [24,33,37-39,42] and 58% at the last follow-up point (n=5) [24,38,39,42].

## Discussion

### Principal Findings

In this review, we described the engagement strategies applied to web-based mindfulness programs and their impact on adherence rates. The use of program features only was associated with program adherence but not with maintaining study retention. Engagement strategies were largely reminders to use the program and, to a lesser extent, the ability to customize program content, interact with features, or engage with content on a deeper level through reflections, homework activities, and discussions of content with facilitators. There was little difference between the type of engagement strategy used and adherence to programs or retention rates.

The need to accurately report study and program attrition to better understand the associations between program adherence and health outcomes has been established [11,12]. Our review found variability across studies in adherence measurements and inconsistencies in reporting adherence. Some studies measured adherence as completing a specific percentage of the program [3,14,30,37,39,41]. Other studies described adherence by grouping the number of sessions completed [5,33,36,38,40,42] or by describing use [24,29,31,32,34,35]. Although findings suggest that program adherence is similar between interventions using program features only and those using a combination of program features and facilitator-led strategies, these results should be interpreted with caution because of the variability in reporting. The variability in measuring adherence is consistent in the e-therapy literature [44] and limits the ability to assess the relationships between adherence to and engagement with web-based interventions and user outcomes. Future studies should consider reporting adherence as a percentage of program completion for easier comparisons across studies.

Similarly, the ability to measure the impact of engagement strategies on study attrition is limited. The findings suggest that studies using only facilitator-led strategies were favorable for maintaining study retention [31,34-36,43]. On average, at the postintervention measurement, studies with only facilitator-led strategies had a retention rate of 93% (SD 10%) compared with the rate of those using only program features of 67% (SD 15%). Similar findings were observed during the follow-up period (76%, SD 7% vs 53%, SD 18%a). However, there is limited evidence as to whether the presence of the facilitator was the reason for this variability or whether other factors such as intervention length, follow-up length, or demographic characteristics of participants contributed to attrition. For example, 1 study that used only program features to improve engagement had low retention after intervention (35%) and at the 8-week follow-up (20%) [24]. No information was provided regarding the reason for these high attrition rates, making it difficult to determine the cause of these findings. The use of a facilitator or therapist to guide web-based psychological programs has been debated [45,46]. Studies of cognitive behavioral therapy interventions found that the presence of a therapist as a facilitator improved symptoms of depression compared with interventions with no facilitator [46]. However, improvements in anxiety symptoms were similar across studies [46], and no information was provided about whether the presence of a facilitator affected adherence. Improvements in patient outcomes may also be explained by the presence of comorbidities, including physical and mental illnesses, on which mindfulness may have a positive impact [47]. Therefore, participation in a mindfulness program targeting 1 disease may have additional benefits for other comorbid conditions. Furthermore, studies that used only facilitator-led strategies experienced, on average, a higher retention rate, which is similar to previous reviews that have described that self-directed interventions often require low levels of support from facilitators [16]. The use of facilitators to encourage adherence, or therapists to deliver content, needs to be weighed against the sustainability goals, cost of the program and length of the intervention during trials, and potential scaling after implementation.

Most studies in this review showed that web-based mindfulness resulted in improvements in either psychological or physiological outcome measures [3,5,14,24,29-34,36,37,39-43]. Two key findings from this review further highlighted the relationship between study retention and baseline functioning of participants, where those with poorer psychological well-being at baseline were more likely to drop out [39], and those with higher adherence were more likely to experience greater improvements in outcomes [30,36,40,42]. This is similar to previous findings where higher levels of worry and rumination at baseline resulted in disengagement from mindfulness-based interventions [23]. Stricter measurements of adherence are required in future studies to fully understand the role of adherence in the success of interventions.

Program features applied throughout studies to enhance engagement varied according to the type and number of features available to users. Furthermore, the number and type of features included had similar impacts on program adherence and study retention, suggesting that there may not be one superior feature to be included in programs. Features such as diaries, reminders, and social connectedness are commonly used in interventions as behavior change techniques [21], and web-based features have been shown to be successful in improving user outcomes in other e-interventions [48]. Within mindfulness, more specific reporting is needed to assess how often users engage with each type of feature to determine the relationship among engagement strategies, adherence, and outcomes.

### Limitations

Across studies, there was a large variance in interventions and in reporting adherence. These factors made it difficult to draw any firm conclusions from the data.

The sample of the included studies was predominately White and female, which limits the generalizability of these findings to other population groups.

This review aims to describe the influence of engagement strategies on adherence and retention among people living with chronic illnesses or conditions. Other studies measuring adherence to mindfulness in the general population may have provided additional information on the impact of engagement strategies. However, there is a need to evaluate engagement and adherence to web-based interventions, specifically in people living with chronic illness. People with chronic illness may be more likely to experience depression and anxiety symptoms than those without a chronic illness [49]. Lower mental well-being can affect the use of and engagement with web-based interventions.

Furthermore, the primary outcome of the review was to assess adherence, retention, and engagement strategies rather than to draw conclusions about the effectiveness of interventions on patient outcomes. As a result, the risk of bias assessment was less relevant.

### Conclusions

Engagement strategies in web-based mindfulness programs largely comprise reminders to use the program. The impact of other features such as personalization, self-reflection activities, and lesson summaries on adherence requires further investigation. There is variance in the way adherence is measured, and intervention lengths and follow-up periods are inconsistent. More thorough reporting and a standardized framework for measuring adherence are needed to more accurately assess adherence and engagement strategies.

## Appendix

Multimedia Appendix 1 Study demographics, examples of search strategy, and adherence, retention, and engagement strategies and outcomes.

## Abbreviations

RCT: randomized controlled trial
