# Supplementary material for: Engagement Strategies to Improve Adherence and Retention in Web-Based Mindfulness Programs: Systematic Review
Source: J Med Internet Res. 2022 Jan 12;24(1):e30026. doi: 10.2196/30026 (PMC8792770; doi:10.2196/30026)
Supplement: Multimedia Appendix 1 [file jmir_v24i1e30026_app1.docx]

Multimedia Appendix - Table 1. Example of search strategy applied to the Medline database

| #70 | #11 AND #29 AND #71 |
| --- | --- |
| #71 | #30 OR #31 OR #32 OR #33 OR #34 OR #35 OR #36 OR #37 OR #38 OR #39 OR #40 OR #41 OR #42 OR #43 OR #44 OR #45 OR #46 OR #47 OR #48 OR #49 OR #50 OR #51 OR #52 OR #53 OR #54 OR #55 OR #56 OR #57 OR #58 OR #59 OR #60 OR #61 OR #62 OR #63 OR #64 OR #65 OR #66 OR #67 OR #68 OR #69 OR #70 |
| #70 | AB retention AND TI retention |
| #69 | AB attrition AND TI attrition |
| #68 | AB engage* AND TI engage* |
| #67 | AB usage ANT TI usage |
| #66 | AB "moderated forum" ANT TI "moderated forum" |
| #65 | AB "chat room" AND TI "chat room" |
| #64 | AB "discussion board" AND TI "discussion board" |
| #63 | AB "social networking" AND TI "social networking" |
| #62 | AB goal* AND TI goal* |
| #61 | AB "goal setting" AND TI "goal setting" |
| #60 | AB “life goal*” AND TI “life goal*” |
| #59 | AB “personal goal*” AND TI “personal goal*” |
| #58 | AB “personal project*” AND TI “personal project*” |
| #57 | AB “personal striving*” AND TI “personal striving*” |
| #56 | AB “life task*” AND TI “life task*” |
| #55 | AB “goal pursuit" AND TI “goal pursuit" |
| #54 | AB “goal achievement*” AND TI “goal achievement*” |
| #53 | AB behavior AND TI behavior |
| #52 | AB "behavior change" AND TI "behavior change" |
| #51 | AB "behavior change taxonomy" AND TI "behavior change taxonomy" |
| #50 | AB “behavior change theory" AND TI “behavior change theory" |
| #49 | AB adherence AND TI adherence |
| #48 | AB diaries AND TI diaries |
| #47 | AB diary OR TI diary |
| #46 | AB "progress track*" AND TI "progress track*" |
| #45 | AB “self-management" AND TI “self-management" |
| #44 | AB “self-help” AND TI “self-help” |
| #43 | AB participat* AND TI participat* |
| #42 | AB habit AND TI habit |
| #41 | AB “habit-form*” AND TI “habit-form*” |
| #40 | AB “form* of habits” AND TI “form* of habits” |
| #39 | AB “self-efficacy" AND TI “self-efficacy" |
| #38 | AB “post-traumatic growth” AND TI “post-traumatic growth” |
| #37 | MH “social networking” |
| #36 | MH “online social networking” |
| #35 | MH goals |
| #34 | MH behavior |
| #33 | MH “treatment adherence and compliance" |
| #32 | MH diary |
| #31 | MH “self-management” |
| #30 | MH “self-care” |
| #29 | #12 OR #13 OR #14 OR #15 OR #16 OR #17 OR #18 OR #19 OR #20 OR #21 OR #22 OR #23 OR #24 OR #25 OR #26 OR #27 OR #28 |
| #28 | AB online AND TI online |
| #27 | AB web AND TI web |
| #26 | AB “web-based” AND TI “web-based” |
| #25 | AB internet AND TI internet |
| #24 | AB “social media” AND TI “social media” |
| #23 | AB app AND TI app |
| #22 | AB “smartphone app” AND TI “smartphone app” |
| #21 | AB “mobile app” AND TI “mobile app” |
| #20 | AB smartphone AND TI smartphone |
| #19 | AB “m-health” AND TI “m-health” |
| #18 | AB “e-health” AND TI “e-health” |
| #17 | AB “internet-based intervention” AND TI “internet-based intervention” |
| #16 | MH “internet-based intervention” |
| #15 | MH internet |
| #14 | MH “social media” |
| #13 | MH “mobile application” |
| #12 | MH smartphone |
| #11 | #1 OR #2 OR #3 OR #4 OR #5 OR #6 OR #7 OR #8 OR #9 OR #10 |
| #10 | AB mindfulness AND TI mindfulness |
| #9 | AB meditation AND TI meditation |
| #8 | AB "mindfulness-based intervention" AND TI "mindfulness-based intervention" |
| #7 | AB "mind-body" AND TI "mind-body" |
| #6 | AB “psychological intervention” AND TI “psychological intervention” |
| #5 | AB “psycho-oncology” AND TI “psycho-oncology” |
| #4 | MH mindfulness |
| #3 | MH meditation |
| #2 | MH “mind-body therapies” |
| #1 | MH “psycho-oncology” |

Table 2. Study demographics

| **Authors** | **Year** | **Country** | **Design** | **Condition** | **Modality** | **Primary outcomes** | **Other outcomes** | **N=** | **% Female** | **Mean age** | **Ethnicity/Race** | **Intervention duration** | **Follow up period** | **Prior mindfulness experience** | **Recruitment method** | **Financial compensation** |
| --- | --- | --- | --- | --- | --- | --- | --- | --- | --- | --- | --- | --- | --- | --- | --- | --- |
| Bostock Crosswell, Prather & Steptoe [36] | 2019 | UK | RCT | Stress | App | Not reported (NR) | Wellbeing Psychological distress Job strain Workplace social support Mindfulness | 238  IG:128 CG:110 | 59 | 36 | NR | 8 weeks (45 sessions) | Post intervention (9-11 weeks)  16 weeks | NR | Online registration | Paid access to app |
| Chandler et al., [5] | 2020 | USA | RCT | Hypertension | App | Resting systolic blood pressure | Blood pressure control (systolic and diastolic Resting diastolic blood pressure Program adherence Perceived stress | 30  IG:16 CG:14 | IG 51 CG 51 | 47 IG 43 CG | NR | 12 months (2 sessions per day) | 1 months 3 months 6 months  12 months | NR | Flyers and clinical referrals | None |
| Compen et al., [37] | 2018 | Netherlands | RCT | Cancer | Web | Distress (Hospital Anxiety Distress Scale) | Fear of cancer recurrence Rumination QOL Mindfulness skills Neuroticism | 245  IG1:90  IG2: 77 CG:78 | 86 | 52 | NR | 8 weeks (weekly session) | 2 months | Experience excluded | Clinicians, online media, offline media, patient associations and peers | None |
| Gotink et al., [14] | 2017 | Netherlands | RCT | Heart disease | Web | 6-minute walk test | Blood pressure Respiratory rate Heart rate Blood sampling and hair cortisol markers QOL Anxiety Depression Perceived stress Social support | 324  IG:215 CG:109 | IG 44 CG 51 | IG: 43 CG: 43 | NR | 12 weeks | 3 months 12 months | IG 14%  CG 13% | Outpatient clinic | None |
| Hearn & Finlay [39] | 2018 | UK | RCT | Spinal cord injury | Web | Depression severity | Pain Anxiety QOL Mindfulness Retention | 67  IG:36 CG:31 | 54 | 44 | 86% white | 8 weeks  (12 sessions a week = 960 minutes) | 2 months 5 months | Experience excluded | Local media invitation letters. Recruitment through clinicians | None |
| Henriksson, Wasara & Rönnlund [40] | 2016 | Sweden | RCT | Chronic pain | Web | NR | Pain Mindfulness Life satisfaction | 107  IG:55 CG:52 | 93 | 51 | NR | 8 weeks  (Twice daily practice for total of 16 hours. 8 "steps" to be completed.) | 8 weeks | Not excluded | Primary care settings, clinics and online | None |
| Huberty et al., [29] | 2019 | USA | RCT | Stress | App | Stress Mindfulness Self-compassion | Sleep disturbance Alcohol consumption Physical activity Fruit and vegetable consumption Acceptability Feasibility | 109  IG:56 CG:53 | 88 | IG 20 CG 22 | 75% non-Hispanic  55% white 17% Asian/Asian American | 8 weeks (daily practice for 10 minutes) | 2 months 3 months | Experience excluded | Social media, email lists, recruitment flyers and through university professors | $5 for baseline survey $10 for 2-month survey $15 for 3-month survey |
| Kladnitski et al., [38] | 2020 | Australia | RCT | Mental health | Web | Depression Anxiety | Distress Functional impairment Treatment credibility Expectancy of benefit | 158  IG1:39 IG2:40 IG3:40 CG:39 | 86 | 39 | NR | 14 weeks (6 lessons) | 14 weeks  26 weeks | Not excluded | Social media and waiting list of people interested in research | None |
| Kubo et al., [30] | 2019 | USA | Pilot RCT | Cancer patients and caregivers | App | NR | Retention Adherence Distress Anxiety Depression Pain Sleep Quality QOL Fatigue Post traumatic growth Mindfulness | 128  Patients IG:54 CG:43  Caregivers IG: 17 CG: 14 | Patients 68  Caregivers 58 | Patients IG: 59 CG: 57  Caregivers  IG: 57 CG: 58 | Patients 63% white  Caregivers 77% white | 8 weeks (daily practice) | 2 months | Experience excluded | Clinician referrals, brochures at clinics, invitation emails | $40 and a year's subscription to Headspace |
| Lindsay et al., [31] | 2018 | USA | RCT | Stress | App | Stress reactivity outcomes | Adherence Treatment expectancies Salivary cortisol Blood pressure Stress | 153  IG1:58 IG2:58 CG:37 | 67 | 32 | Non-Hispanic or Latino 95%  White 53% Asian 22% Black/African American 22% | 2 weeks (daily practice) | 2 weeks | Prior and regular practice excluded | Research registries, community advertisements and emails to local organizations | Yes, not specified |
| Moberg, Niles & Beermann [24] | 2019 | USA | RCT | Mental health | App | NR | Depression Anxiety Stress Self-efficacy App usage Moderator measures | 500  IG: 253 CG: 247 | IG: 75 CG: 74 | IG: 30 CG: 30 | White 82% | 1 month (daily practice) | 1 month 3 months | Pacifica users excluded | Social media, opt in ads within app | None |
| Rosen et al., [32] | 2018 | USA | RCT | Cancer | App | QOL | Mindfulness Pain Health literacy App usage | 112  IG:57 CG:55 | 100 | 52 | Non-Hispanic 93%  White 86% | 8 weeks (10 days minimum requirement) | 5 weeks 9 weeks  3 months | Not excluded | Presentations, flyers, letters to colleagues. Online through social media, message boards, blogs and research registries | Free access to Headspace for 6 months ($77.94) |
| Russell et al., [3] | 2019 | Australia | Pilot RCT | Cancer | Web | NR | Acceptability Engagement Fear of cancer recurrence Rumination Worry Mindfulness Perceived stress | 69  IG:46 CG:23 | 54 | 54 | NR | 6 weeks (weekly lesson and twice daily practice, 70 minutes per week) | 6 weeks | Not excluded 38% reported previous experience | Clinic | None |
| Stjernsward & Hansson [27] | 2018 | Sweden | RCT | Caregivers of people with mental or somatic illness | Web | Mindfulness | Self-Compassion Perceived stress Burden Usability Confounding factors  Negative effects of training | 398  IG:196  CG:202 | 86 | 53 | NR | 8 weeks (weekly lesson and daily practice for 6/7 days - 120 minutes per week) | 2 months 5 months | Experience excluded | Advertisements in papers newsletters, online, social media and in clinics and organizations | None |
| Tavallaei, Rezapour-Mirsaleh, Rezaiemaram & Saadat [43] | 2018 | Iran | RCT | Chronic headache | Web | NR | Distress Pain Disability Mindfulness | 30  IG:15 CG:15 | 100 | IG: 32 CG: 35 | NR | 8 weeks | 8 weeks | Regular mediation or yoga excluded | Clinic | None |
| Thompson et al., [33] | 2015 | USA | RCT | Depression | Web | Depression | Knowledge and skills Depression Coping Self-efficacy Self compassion  Life satisfaction QOL | 118  IG:62 CG:56 | 65 | 41 | Non-Hispanic 98%  White 59% | 8 weeks (Weekly hour sessions) | 9-10 weeks 18-20 weeks | NR | University clinics | $15 for each weekly session attended $25 for each completed assessment |
| Wahbeh, Goodrich & Oken [35] | 2016 | USA | Pilot RCT | Stress | Web | NR | Feasibility Acceptability Mood Cognitive outcomes | 21  IG:8  CG:8 | 50 | 76 | White 88% | 6 weeks (One-hour weekly sessions and 30 minutes daily practice) | 6 weeks | Practice in last 6/12 excluded | Informational talk, community flyers and clinician referrals | None |
| Wahbeh [34] | 2018 | USA | RCT | Depression | Web | Depression | Spiritual experiences Resilience Mindfulness Satisfaction Pain Perceived stress | 50  IG:26 CG:24 | 80 | 65 | White 84% | 6 weeks (One-hour weekly sessions and 20-30 minutes daily practice) | 6 weeks 12 weeks | Practice in last 6/12 excluded | Community networks, online listservs and postings, community flyers, housing, and social groups | None |
| Younge et al., [41] | 2015 | Netherlands | RCT | Heart disease | Web | 6-minute walk test | Weight Blood pressure Respiratory rate  Heart rate Blood sampling Subjective health status Psychological wellbeing Stress Social support Adverse events | 324  IG:215 CG:109 | 46 | 43 | NR | 12 weeks | 3 months | Not excluded | Clinic | None |

NR = not recorded, RCT = randomized controlled trial, IG intervention group, CG = control group, AC = active control, 6/12 = six months, QOL = Quality of Life

Table 3. Adherence, retention, engagement strategies and outcomes

|  | **Adherence** | | | | | **Retention** | | **Engagement strategies** | | **Outcome measures** | |
| --- | --- | --- | --- | --- | --- | --- | --- | --- | --- | --- | --- |
|  | **Adherence defined** | **How adherence was measured** | **Definition of adherence as a percentage** | **Definition of adherence when grouped** | **Adherent with intervention protocol**  **(%)** | **Retention at post intervention (%)** | **Retention at last follow up**  **(%)** | **Program features** | **Facilitator-led strategies** | **Primary findings** | **Relationship between adherence and outcomes** |
| Bostock Crosswell, Prather & Steptoe [36] | Y | 25-45 sessions (n=35, 27%) 10-24 sessions (n=52, 4%) <10 sessions (n=41, 32%) | NR | 55-100 | 27 | 96 | 82 | NR | Weekly reminder email from research staff with encouragement to use the program | Mean sessions complete 16.6. 2% completed 100% of program Number of sessions completed was positively correlated with age (p=0.002) Intervention group had significantly improved wellbeing, daily positive emotions, anxiety, depression, job strain and workplace social support (p <0.05) Completing more than 10 sessions resulted in greater improvements in wellbeing, daily positive affect, anxiety, and depression (p<0.05) Improvements in wellbeing job strain and depression were sustained to 16 weeks | Completing more than 10 sessions resulted in greater improvements in wellbeing, daily positive affect, anxiety, and depression (p<0.05) |
| Chandler et al., [5] | Y | Percentage meeting 75% cut-off 1 month 60% 3 months 64% 6 months 58% 12 months 39% | NR | 75 | 39 | 81 | NR | Immediate post mediation heart rate feedback chart, text messages to encourage motivation and social reinforcement tailored to adherence rates. In session prompts to guide practice e.g., phone detects movement and displays a message to re-focus on practice.  Ability to personalize app - turn off audio guide, change background images and set session reminders | Opportunity to ask questions of research team after first session | The intervention group had higher percentage of systolic blood pressure control at 6 and 12 months (p<0.02)  In the intervention group there were larger reductions in systolic and diastolic blood pressure at 3, 6 and 12 months. | NR |
| Compen et al., [37] | Y | Adherence defined as at least 4 sessions completed (50% of program).  IG1: 91% started sessions 79% completed 4 or more sessions  IG2: 91% started sessions 92% completed 4 or more sessions | 50 | NR | 79 | 70 | NR | Participants encouraged to fill out practice diaries each day and asked to complete an essay about their experiences of a "silent day". | Therapist provided written feedback on all diaries and essays. | Participants in IG1 and IG2 had significantly improved distress, fear of cancer recurrence, rumination, and mental health related QOL at 8 weeks compared to CG.  Participants in IG1 and IG2 had better mindfulness skills and increased positive mental health compared to CG.  Participants in IG1 or IG2 with higher baseline neuroticism showed larger improvements in distress than CG. | NR |
| Gotink et al., [14] | Y | 50% were adherent and completed at least 50% of the program. | 50 | NR | 50 | NR | 74 | Bi-weekly email reminders and text messages for the 12-week intervention period and up to 12 months follow up to encourage practice. | NR | Cohen's d showed significant improvement in 6MWT (d-0.22), improvements in systolic blood pressure (d=0.19), mental functioning (d=0.22) and depression (d=0.18). | Those with higher blood pressure (p=0.031) were more likely to be compliant with the intervention. |
| Hearn & Finlay [39] | Y | Adherence was measured as 100% completion of content.  72% completed the whole program.  Non-completers viewed on average 217 minutes of program content. | 100 | NR | 72 | 72 | 58 | Videos were used to demonstrate to participants how to adopt mindfulness practices using small movements for people with reduced physical function. | NR | At 2 months the intervention group showed significant improvements in depression, anxiety, pain unpleasantness and pain catastrophizing (p<0.05). There were significant improvements in the following aspects of mindfulness: acting with awareness, non-judging, non-reactivity to inner peace and total mindfulness score.  Improvement in depression, anxiety, and pain catastrophizing were sustained to 3 months (p<0.05) | People with higher depression scores at baseline were more likely to drop out than those with lower depression scores p=0.051 |
| Henriksson, Wasara & Rönnlund [40] | Y | 50% completed the program. 58% completed 88% + of the program. 25% completed at least half and 17% completed less than half | NR | 88-100 | 58 | 66 | NR | Email reminder to encourage and remind participants to use the program. | NR | IG showed higher mindfulness scores at 8 weeks compared to baseline and compared to the CG.  IG had lower pain and pain severity at 8 weeks compared to CG and had reduced pain severity between baseline and 8 weeks follow up.  Pain interference was improved in the IG over time. Those with high adherence (completed 7 or more weeks) had improved pain suffering over time and between groups, and improved pain distress over time.  Life satisfaction was improved in the IG between groups and over time. Life control was improved in the IG over time. | Those with high adherence (completed 7 or more weeks) had improved pain suffering over time and between groups, and improved pain distress over time. |
| Huberty et al., [29] | N | 56% completed more than 30 minutes of meditation per week, of that 22% completed more than 60 minutes a week. 34% continued meditation during follow up | not defined | not defined | NR | 79 | 59 | Participants could select own meditation program to suit their individual goals Participants who meditated less than 30 minutes a week were sent a text message reminder to meditate | NR | IG showed significant reductions in stress at 2 months (p<0.001), improvements in mindfulness (p<0.001) and self-compassion (<0.0001).  Positive improvements in stress, mindfulness and self-compassion were sustained to 3 months in the IG.  51% felt the Calm app was helpful 85% were satisfied with the app and enjoyed using it. 68% were extremely likely to use Calm in the future 76% would recommend Calm to others | NR |
| Kladnitski et al., [38] | Y | IG1: 68% completed 100% 83% completed 75%  IG2: 66% completed 100% 81% completed 75%   IG3: 66% completed 100% 66% completed 75% | NR | 100 | 66 | 77 | 83 | Lesson summaries of key concepts and homework activities including exercises, diaries and monitoring forms. | After lesson 2 email contact with clinician to discuss experience provide support or phone consultation.  Email support initiated in response to a request from participants, for high distress, depression or suicidal thought scores or if there was no participation on the site in more than 10 days. | Within group effects showed significant improvements in anxiety and depression over time (p<0.001). IG2 showed a small but non-significant positive effect across all measures in comparison to IG1 and IG3.  in IG1 and IG2 there was no clinical deterioration in anxiety or depression observed in any participant. | No significance differences in baseline scores between completers and non-completers |
| Kubo et al., [30] | Y | 50% (patients) and 62% (caregivers) practised mindfulness for 50% of the days across the 8 weeks.  33% (patients) and 39 (caregivers) practised for at least 70% of the days.  70% used the app after the study period | 50 | NR | 50 & 62 | 74 | NR | Participants could elect to receive push notification reminders. Participants could select courses to complete to meet their own conditions or situations | Staff contacted participants by phone if they completed fewer then 3 sessions each week. | Patients IG has significantly improved wellbeing and emotional wellbeing (p=0.03). Those with 50% adherence had greater improvements in emotional wellbeing, pain interference, depression, spiritual change, non-judgement of inner experience, and overall wellbeing (all p<0.05) Caregivers Ig had significantly improved observing mindfulness scores (p=0.03) and overall posttraumatic growth (p=0.05) | Participants who practiced mindfulness on 50% or more of the days showed significantly higher emotional wellbeing, pain, depression, spiritual change, non-judgement of inner experience and overall wellbeing (all p<0.05) |
| Lindsay et al., [31] | N | 75% completed 100% of the program Mean completion of 13.49 sessions out of 14. | not defined | not defined | NR | 95 | NR | NR | Staff contacted participants by phone on day 3 and 9 to answer training specific questions, address difficulties and encourage adherence | IG1 had significantly lower cortisol than IG2 and CG at follow-up during an induced stress test (p=0.037 and p=0.03 respectively). IG1 had significantly lower systolic blood pressure than IG2 and CG at follow-up during an induced stress test (p=0.037 and p=0.029 respectively). | NR |
| Moberg, Niles & Beermann [24] | N | Median logins 19.  Use of thoughts record tool was significantly associated with anxiety and stress. | not defined | not defined | NR | 35 | 20 | Participants selected goals to work towards, were asked to rate mood each day and were provided with prompt resources for mood depending on their score. Participants had the ability to track their health behaviors (diet, exercise, sleep), personal customizable inspiration board, peer discussion board, progress tracking. | NR | IG had significant improvements in depression, anxiety, stress and self-efficacy at 1 month follow up.  IG showed improvements in depression, anxiety, stress and self-efficacy between baseline and 1-month follow up. IG had a clinically significant change in depression (p<0.0001) and anxiety (p=0.031). IG showed benefits of the interventions were significantly higher when analyzing for stress for those not taking psychiatric medication compare to those taking medication, and those not taking medication had significant reductions in anxiety. | No impact of mindfulness of depression, anxiety, stress or self-efficacy. Those who used though record tools had significantly lower anxiety and stress |
| Rosen et al., [32] | N | Mean logins were 18 days of 8-week period. Mean duration of use 13.4 minutes per day | not defined | not defined | NR | NR | 54 | After completing a standardized 10-day course, participants could choose which course to follow based on their own preferences and goals.  Weekly email or text reminders to encourage participation | NR | IG had improved QOL at 3 months compared to CG (p<0.01) and higher mindfulness scores (p=0.04) | Higher QOL scores at baseline was positively associated with completion (p=0.002) and those with less pain at baseline were more likely to complete the intervention (p=0.013). |
| Russell et al., [3] | Y | Adherence to meditation ranged from 61% (Week 2) to 80% (Week 4)  70 minutes required for weeks 1-2 140 minutes required for week 3-6 | 100 | NR | NR | 70 | NR | Participants received twice daily email reminders to practice mindfulness | NR | IG had significantly lower severity of fear of cancer recurrence at 6 weeks (p=0.008). 72% found the program helpful | NR |
| Stjernsward & Hansson [27] | Y | 2 months 16% used the program 0-120 minutes 27% practiced 121-480 minutes 57% practiced 481-960 minutes  3 months 21% used the program 0-120 minutes 22% practiced 121-480 minutes 57% practiced 481-960 minutes |  | 50-100 | 57 | 69 | 49 | Participants had a private diary that they could complete. Participants received on screen reminder to complete informal practice. | Weekly email reminders from staff for technical and contact reassurance and to encourage adherence. | IG had significantly improved mindfulness (p=0.001), self-compassion (p=0.001), stress (p=0.001) compared to CG. Mean usability was low 21 (>70 represents good usability) | Those who practiced more mindfulness exercises had higher mindfulness scores at post-intervention and follow-up. |
| Tavallaei, Rezapour-Mirsaleh, Rezaiemaram & Saadat [43] | N | Not reported | not defined | not defined | NR | 100 | NR | NR | Weekly follow up by therapist to discuss exercise and to answer concerns. Phone calls lasted 30 minutes each week | There were significant changes in pain, distress, disability and mindfulness (all p<0.05) | NR |
| Thompson et al., [33] | Y | Mean sessions attended was 6.6. Adherence was measured as 0-4 sessions, 5-7 sessions and 8 session completed. | NR | 100 | NR | 80 | 80 | Weekly check-in, group discussion, skills building exercises and homework. | Participants were called or sent a reminder email if they had not viewed new weekly content within 3 days. Participants were telephoned if they missed a session | IG showed significant improvements in satisfaction (p=0.006), knowledge and skills (p=0.016), and depression (p<0.05) | NR |
| Wahbeh, Goodrich & Oken [35] | N | Mean sessions completed 4.2 with 604 minutes of practice at home and 21 days of practice. | not defined | not defined | NR | 100 | NR | NR | Weekly telephone calls from staff to encourage use and answer any concerns. | There was no significant change in any outcome measures (all p>0.05) | NR |
| Wahbeh [34] | N | Mean sessions completed 3.2 with 590 minutes of practice at home and 16 days of practice over 6 weeks. | not defined | not defined | NR | 73 | 69 | NR | Weekly telephone calls from staff to encourage use and answer any concerns. | IG showed improvements in depression, stress, sleep disturbance and pain severity by 7 weeks (p<0.05) compared to the waitlist group.  Pe-post effect showed that all participants (In IG and waitlist) showed significant improvements in depression, stress, sleep disturbance, pain interference and spirituality (p<0.05) Satisfaction with program mean 2.5/4 Improvements were sustained to 14 weeks for depression, stress, sleep disturbance, pain interference and spirituality (p<0.05) | No significant relationship between program use (number or duration of sessions) and outcome measures. |
| Younge et al., [41] | Y | Adherence measured as 50% completed. 53% completed at least 50% of the program. | 50 | NR | 53 | 78 | NR | Bi-weekly reminders by email and text message | NR | IG showed significant improvements in 6 minute walk test (p=0.05), heart rate (p=0.033).  Cohen's d showed significant improvements in heart rate and depression. As-treated analysis showed significant improvements in 6 minute walk test, heart rate, systolic blood pressure and stress. | NR |
| NR = not reported, CG = control group, IG = intervention group | | | | | | | | | | | |

Table 4. Engagement strategies used within program

| **Authors** | **Personalize content/ reminder** | **Personalize appearance** | **Self-reflection** | **Goals** | **Immediate feedback** | **Social** | **Progress practice tracking** | **Homework activities** | **Demonstrations** | **Lesson summaries** | **Psychological tracking** | **Physical health tracking** | **Program reminders** | **None** | **Number of strategies used** | **Adherence (%)** | **Retention post intervention** | **Retention last data collection point** |
| --- | --- | --- | --- | --- | --- | --- | --- | --- | --- | --- | --- | --- | --- | --- | --- | --- | --- | --- |
| Bostock Crosswell, Prather & Steptoe [32] |  |  |  |  |  |  |  |  |  |  |  |  |  | 🗸 | 0 | 27 | 96 | 82 |
| Chandler et al., [5] | 🗸 | 🗸 |  |  | 🗸 | 🗸 |  |  |  |  |  |  | 🗸 |  | 5 | 39 | 81 |  |
| Compen et al., [37] |  |  | 🗸 |  |  |  |  | 🗸 |  |  |  |  |  |  | 2 | 79 | 70 |  |
| Gotink et al., [14] |  |  |  |  |  |  |  |  |  |  |  |  | 🗸 |  | 1 | 50 |  | 74 |
| Hearn & Finlay [39] |  |  |  |  |  |  |  |  | 🗸 |  |  |  |  |  | 1 | 72 | 72 | 58 |
| Henriksson, Wasara & Rönnlund [40] |  |  |  |  |  |  |  |  |  |  |  |  | 🗸 |  | 1 | 58 | 66 |  |
| Huberty et al., [29] | 🗸 |  |  |  |  |  |  |  |  |  |  |  | 🗸 |  | 2 | NR | 79 | 59 |
| Kladnitski et al., [38] |  |  |  |  |  |  |  | 🗸 |  | 🗸 |  |  |  |  | 2 | 66 | 77 | 83 |
| Kubo et al., [30] | 🗸 |  |  |  |  |  |  |  |  |  |  |  | 🗸 |  | 2 | 56 | 74 |  |
| Lindsay et al., [31] |  |  |  |  |  |  |  |  |  |  |  |  |  | 🗸 | 0 | NR | 95 |  |
| Moberg, Niles & Beermann [24] |  | 🗸 |  | 🗸 |  | 🗸 | 🗸 |  |  |  | 🗸 | 🗸 |  |  | 6 | NR | 35 | 20 |
| Rosen et al., [32] | 🗸 |  |  |  |  |  |  |  |  |  |  |  | 🗸 |  | 2 | NR |  | 54 |
| Russell et al., [3] |  |  |  |  |  |  |  |  |  |  |  |  | 🗸 |  | 1 | NR | 70 |  |
| Stjernsward & Hansson [27] |  |  | 🗸 |  |  |  |  |  |  |  |  |  |  |  | 1 | 57 | 69 | 49 |
| Tavallaei, Rezapour-Mirsaleh, Rezaiemaram & Saadat [43] |  |  |  |  |  |  |  |  |  |  |  |  |  | 🗸 | 0 | NR | 100 |  |
| Thompson et al., [33] |  |  |  |  |  | 🗸 |  | 🗸 |  |  |  |  |  |  | 2 | NR | 80 | 80 |
| Wahbeh, Goodrich & Oken [35] |  |  |  |  |  |  |  |  |  |  |  |  |  | 🗸 | 0 | NR | 100 |  |
| Wahbeh [34] |  |  |  |  |  |  |  |  |  |  |  |  |  | 🗸 | 0 | NR | 73 | 69 |
| Younge et al., [41] |  |  |  |  |  |  |  |  |  |  |  |  | 🗸 |  | 1 | 53 | 78 |  |
| NR = not recorded | | | | | | | | | | | | | | | |  |  |  |
